# Novel mechanism of cardiac protection by valsartan: synergetic roles of TGF-β1 and HIF-1α in Ang II-mediated fibrosis after myocardial infarction

**DOI:** 10.1111/jcmm.12551

**Published:** 2015-03-30

**Authors:** Xizhong Sui, Hongchao Wei, Dacheng Wang

**Affiliations:** a Department of Cardiothoracic Surgery, The Civil Aviation General HospitalChaoyang District, Beijing, China; b The Wulanchabu Medical CollegeJining, Inner Mongolia, China

**Keywords:** valsartan, myocardial infarction, fibrosis, TGF-β1, Hif-1α

## Abstract

Transforming growth factor (TGF)-β1 is a known factor in angiotensin II (Ang II)-mediated cardiac fibrosis after myocardial infarction (MI). Hypoxia inducible factor-1 (Hif-1α) was recently demonstrated to involve in the tissue fibrosis and influenced by Ang II. However, whether Hif-1α contributed to the Ang II-mediated cardiac fibrosis after MI, and whether interaction or synergetic roles between Hif-1α and TGF-β pathways existed in the process was unclear. *In vitro*, cardiac cells were incubated under hypoxia or Ang II to mimic ischaemia. *In vivo*, valsartan was intravenously injected into Sprague–Dawley rats with MI daily for 1 week; saline and hydralazine (another anti-hypertensive agent like valsartan) was used as control. The fibrosis-related proteins were detected by Western blotting. Cardiac structure and function were assessed with multimodality methods. We demonstrated *in vitro* that hypoxia would induce the up-regulation of Ang II, TGF-β/Smad and Hif-1α, which further induced collagen accumulation. By blocking with valsartan, a blocker of Ang II type I (AT1) receptor, we confirmed that the up-regulation of TGF-β/Smad and Hif-1α was through the Ang II-mediated pathway. By administering TGF-β or dimethyloxalylglycine, we determined that both TGF-β/Smad and Hif-1α contributed to Ang II-mediated collagen accumulation and a synergetic effect between them was observed. Consistent with *in vitro* results, valsartan significantly attenuated the expression of TGF-β/Smad, Hif-1α and fibrosis-related protein in rats after MI. Heart function, infarcted size, wall thickness as well as myocardial vascularization of ischaemic hearts were also significantly improved by valsartan compared with saline and hydralazine. Our study may provide novel insights into the mechanisms of Ang II-induced cardiac fibrosis as well as into the cardiac protection of valsartan.

## Introduction

Myocardial infarction (MI) involves a progression of deterioration towards heart failure, which will lead to the irreversible impairment of cardiac function and is a major cause of mortality. A key event in the deterioration process is the dynamic ventricular remodelling involving cell population changing, especially excessive increase in fibroblasts 1. After MI, lots of viable cells would be replaced by the extracellular matrix (ECM) which is mainly produced from fibroblasts, inducing the deposition of collagen in the myocardium and myocardial fibrosis.

During the production of collagen, ECM regulatory proteins such as matrix metalloproteinases (MMPs) and cytokines such as transforming growth factor (TGF)-β1 directly affect the turnover of ECM 2,3. TGF-β1 in the cardiac fibrosis and ECM remodelling have been addressed intensely 4–6. It has been reported that TGF-β1 influenced the process of cell proliferation, migration and ECM homoeostasis (including synthesis and degradation) 7. After MI, activation of the TGF-β pathway would promote myocardial fibrosis and the formation of scars 4. Thus, TGF-β was considered as a target to regulate the pathological process after MI.

As a blocker in Ang II receptor, the potential effects of valsartan have been widely investigated in the diseases of different organs, including liver fibrosis 8, kidney injury 9 and cardiovascular injury, especially heart failure and cardiac hypertrophy 10–13. It has been found that treatment with valsartan could significantly inhibit the TGF-β pathway and further attenuate the progression of liver fibrosis of the liver 8. In cardiovascular disease, studies also found that valsartan could inhibit LV hypertrophy, dilatation and protect cardiac function 14,15. Hif-1α was recently demonstrated to be involved in tissue fibrosis and influenced by Ang II 16,17. However, whether it contributed to the Ang II-mediated cardiac fibrosis after MI was not investigated. In addition, whether interaction or synergetic roles between Hif-1α and TGF-β pathways existed in myocardial fibrosis after MI was unclear.

In this study, we investigated the potential effects of both the Hif-1α and TGF-β pathways in cardiac fibrosis after MI, and their synergetic roles were explored.

## Materials and methods

### Cultivation of cardiac cells and *in vitro* treatment

We isolated cardiac cells from 0- to 3-day-old neonatal Sprague–Dawley (SD) rats through methods described previously. The fresh isolated cardiac cells were pre-seeded for 2 hrs in DMEM culture medium (Gibco) to remove the fibroblasts; then they were collected (6 × 10^5^/ml) and cultivated in cell plates. To induce hypoxia, 1% O_2_, 5% CO_2_ and 92.5% N_2_ was set for incubated the cardiac cells for 4 hrs and serum was deprived as described previously 18,19. Valsartan (Changzhou Kony Pharm Co., Ltd., Jiangsu, China) was supplemented in the medium and the final concentration of valsartan dissolved in dimethyl sulfoxide (Sigma-Aldrich) was ≤3‰ (v/v) which was incubated for 48 hrs. TGF-1β (5 ng/ml; Sigma-Aldrich) was administered to activate the TGF-1β pathway. The agonist of dimethyloxalylglycine (DMOG; Santa Cruz) was used (1 mM) to induce the accumulation of Hif-1α proteins.

### Model of myocardia infarction and treatment

Exactly, 45 male SD rats weighting 250–300 g were purchased from the Academy of Military Medical Sciences (Beijing, China). Rats were anaesthetized with injection of sodium pentobarbital (30 mg/kg, intraperitoneally) in the abdominal cavity. After thoracotomy, acute MI was induced by ligation of the left coronary artery with prolene suture as described previously 20. Animals were then randomly divided into two groups: (*i*) valsartan-treated group that was given intravenously 3 mg/kg/day valsartan in 0.5 ml normal saline *via* the vein daily for 1 week; (*ii*) hydralazine (Sigma-Aldrich)-treated group receiving 0.2 mg/kg/day hydralazine injection in saline; and (*iii*) control group that received saline injection in the same way (*n* = 15 for each group).

### Determination of cardiac function through echocardiography and haemodynamics

Echocardiography (14.0 MHz, Sequoia 512; Acuson) was used to determine the cardiac function 4 weeks after surgery. LV fractional shortening (LVFS) and LV ejection fraction (LVEF) were measured according to previous reports 21,22.

To further evaluate the effect of valsartan on cardiac function of rats with MI, the arterial and haemodynamic factors were recorded with a catheter-tipped micro-manometer through the insertion of the catheter into the ascending aorta and the LV.

### Histological examination

Rats were killed and the hearts were explanted quickly. The hearts were then embedded in OCT and frozen in liquid nitrogen to prepare the frozen sample. Thereafter, 4-μm frozen sections were prepared for histological examination. To determine infarct size and LV wall thickness, Masson’s trichrome staining was performed. Infarct size and wall thickness of hearts were then determined with a computer-assisted method 21, which was calculated by the percentage of endocardial circumference in the injured endocardial circumference. Five different transversal sections with apparent infarct area were prepared.

### Western blotting assays

Cells and cardiac tissues (infarcted myocardium) were collected and lysed in Laemmli sample buffer (Bio-Rad). The isolated proteins were collected and determined through the BCA protein assay kit (Thermo Scientific). Proteins were then loaded in SDS-PAGE gel and separated through electrophoresis, which was followed by transferring them to a polyvinylidene fluoride (PVDF) membrane (Roche). After blocking with non-fat milk, the membrane was incubated with primary antibodies overnight at 4°C. The unconjugated antibodies were removed by washing and the horseradish peroxidase-conjugated secondary antibodies were incubated for 2 hrs in room temperature. The bands were detected by enhanced chemiluminescence (Applygen). ImageJ software was used to determine the band intensity, which was normalized to the internal standard.

### Immunohistochemical staining

To determine the effect of valsartan on vascularization, the slices of infarct hearts were immunohistochemically stained with von Willebrand factor (vWF; Sigma-Aldrich) antibody, and were then incubated with peroxidase-conjugated streptavidin and diaminobenzidine (DAB). Arteriole density was quantified by choosing five fields in the infarcted area randomly from each section. The arteriole density was expressed as number of arterioles/mm^2^. The vessel diameters were also measured by the computer-assisted method utilizing the ImagePro program.

### Statistical analysis

All data, expressed as mean ± SD, were analysed by Student’s *t*-test or one-way anova with SPSS 17.0 statistics software. *P* < 0.05 was considered to be statistically significant.

## Results

### Hypoxia-induced changes in Ang II and related proteins

**Figure 1:**
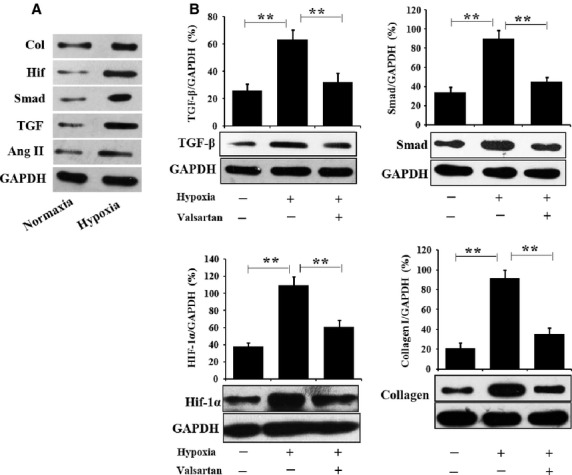
*In vitro* protective effect of valsartan on cardiac cells under hypoxia. (A) Hypoxia treatment up-regulated the expression of Ang II, TGF-1β/Smad, Hif-1α and increased collagen accumulation; (B) Western blotting analysis demonstrated that valsartan, a blocker of the Ang II type I receptor, significantly suppressed hypoxia-upregulated TGF-1β/Smad, Hif-1α and collagen accumulation, indicating that hypoxia-induced up-regulation of TGF-1β/Smad, Hif-1α and collagen accumulation may be mediated by Ang II through its type I receptor (*n* = 3, **P < 0.01).

*In vitro*, cardiac cells were incubated in hypoxia to mimic ischaemia. Under hypoxia, Ang II, TFG-β/Smad and Hif-1α were significantly up-regulated, as shown in Figure1A. Meanwhile, accumulation of collagen was also increased. To determine whether changes in TFG-β/Smad, Hif-1α and collagen under hypoxia were related to Ang II, valsartan was administered to block the AT1 receptor. As shown in Figure1B, the unregulated expression of TFG-β/Smad, Hif-1α and collagen in hypoxia was significantly decreased by valsartan, indicating that hypoxia-upregulated TFG-β/Smad, Hif-1α and collagen expression may be mediated by Ang II through the AT1 receptor.

### Synergetic roles of TGF-β1 and HIF-1α in Ang II-mediated collagen accumulation

**Figure 2:**
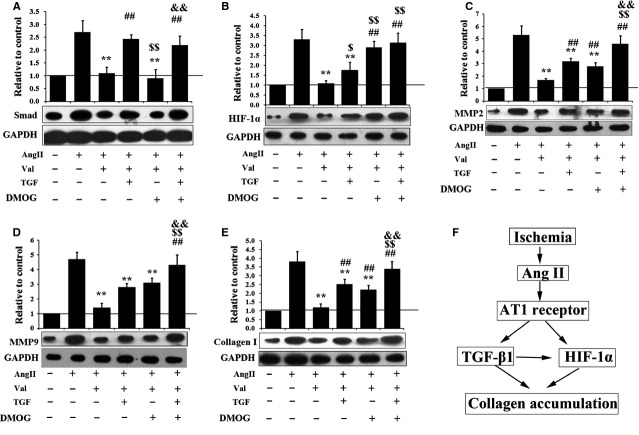
Effects of Ang II, valsartan, TGF-1β and DMOG on TGF-1β/Smad and Hif-1α pathways as well as fibrosis-related proteins. (A) Expression of Smad (downstream effector of TGF-1β) when Ang II, valsartan, TGF-1β or DMOG were added. (B) Expression of Hif-1α when Ang II, valsartan, TGF-1β or DMOG were added. (C) Expression of MMP-2 when Ang II, valsartan, TGF-1β or DMOG were added. (D) Expression of MMP-9 when Ang II, valsartan, TGF-1β or DMOG were added. (E) Collagen accumulation when Ang II, valsartan, TGF-1β or DMOG were added. (F) Regulatory network mapped based on *in vitro* results (*n* = 3, ** compared with Ang II; ## compared with Val+Ang; $$ compared with Ang+val+TGF; && compared with Ang+Val+DMOG).

To confirm whether Ang II-induced collagen accumulation was through TGF-β1/Smad and HIF-1α pathways, TGF-β1 (TGF-β1/Smad pathway) and DMOG (agonist of HIF-1α) were administered together with Ang II and valsartan. As shown in Figure2A, Ang II-upregulated Smad was counteracted by valsartan, when TGF-1β was added; the Smad expression was restored, indicating that Ang II inhibited TGF-1β which further inhibited its downstream pathway. The Ang II-induced Hif-1α expression was also suppressed by valsartan and could be restored by DMOG (Fig.2B). Interestingly, valsartan-suppressed Hif-1α under Ang II could also be partly restored by TGF-1β, indicating Hif-1α may be influenced by the TGF-1β pathway to some extent, but addition of DMOG exerted no effects on TGF-1β/Smad.

We then analysed the expression of fibrosis-related proteins (MMP2, MMP9 and collagen I) to determine the roles of TGF-β1/Smad and HIF-1α pathways in myocardial fibrosis. As shown in Figure2C–E, Ang II-induced up-regulation of MMP2, MMP9 and collagen I was suppressed by valsartan, which is consistent with previous reports that Ang II mediated myocardial fibrosis through its AT1 receptor. Adding TGF-β1 or DMOG alone partly restored the expression of MMP2, MMP9 and collagen I. When TGF-β1 and DMOG were added together, the expression of MMP2, MMP9 and collagen I was further up-regulated, comparable with that before valsartan addition, indicating that both TGF-β1 and DMOG pathways participated in Ang II-mediated myocardial fibrosis, and suggesting the presence of synergistic effects. The relative roles of TGF-β1 and HIF-1α pathways in Ang II-induced myocardial fibrosis after MI are mapped in Figure2F.

### Suppression of fibrosis-related proteins by valsartan after MI

**Figure 3:**
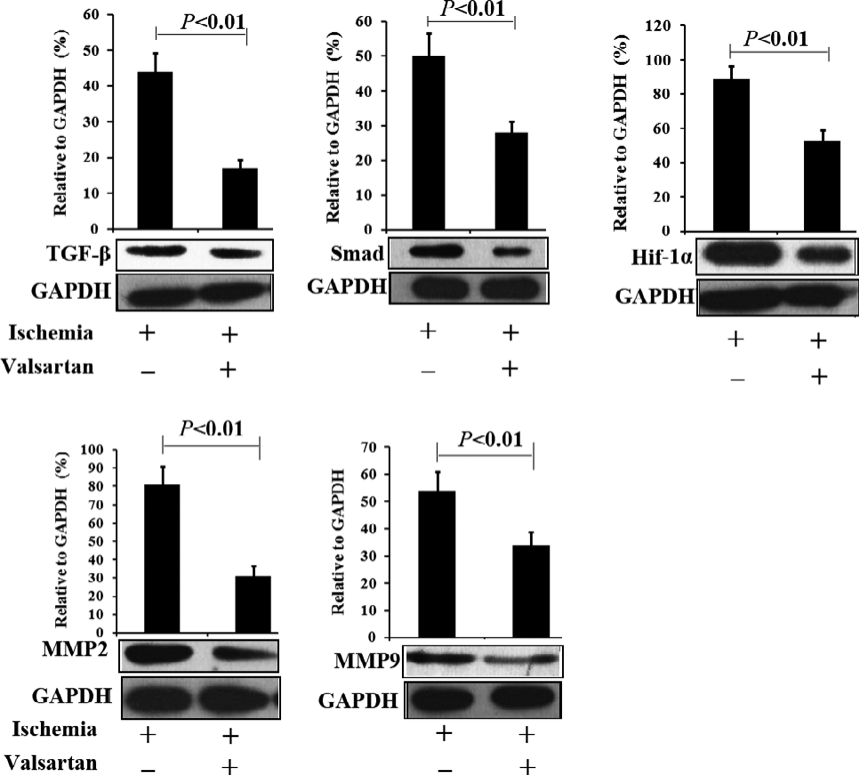
Influence of valsartan on myocardial expression of TGF-1β/Smad, Hif-1α and fibrosis-related proteins 1 week after MI. Compared with animals of MI, administering valsartan significantly decreased the expression of TGF-1β/Smad, Hif-1α and fibrosis-related proteins (MMP-2 and MMP-9) which were up-regulated after MI. Ischaemia indicates animals with MI and receiving saline treatment. Valsartan indicates animals receiving valsartan treatment (*n* = 5).

To validate the cardiac protective effects of valsartan after ischaemia, a rat model of MI was prepared and valsartan was administered. At 1 week, hearts were explanted and Western blotting was performed. As shown in Figure3, TGF-β1/Smad and HIF-1α pathways were up-regulated after MI, but they were significantly suppressed by the administration of valsartan. MI-induced up-regulation of fibrosis-related proteins; MMP2 and MMP9 were also suppressed by valsartan, indicating that valsartan may attenuate myocardial fibrosis after MI, consistent with *in vitro* results.

**Figure 4:**
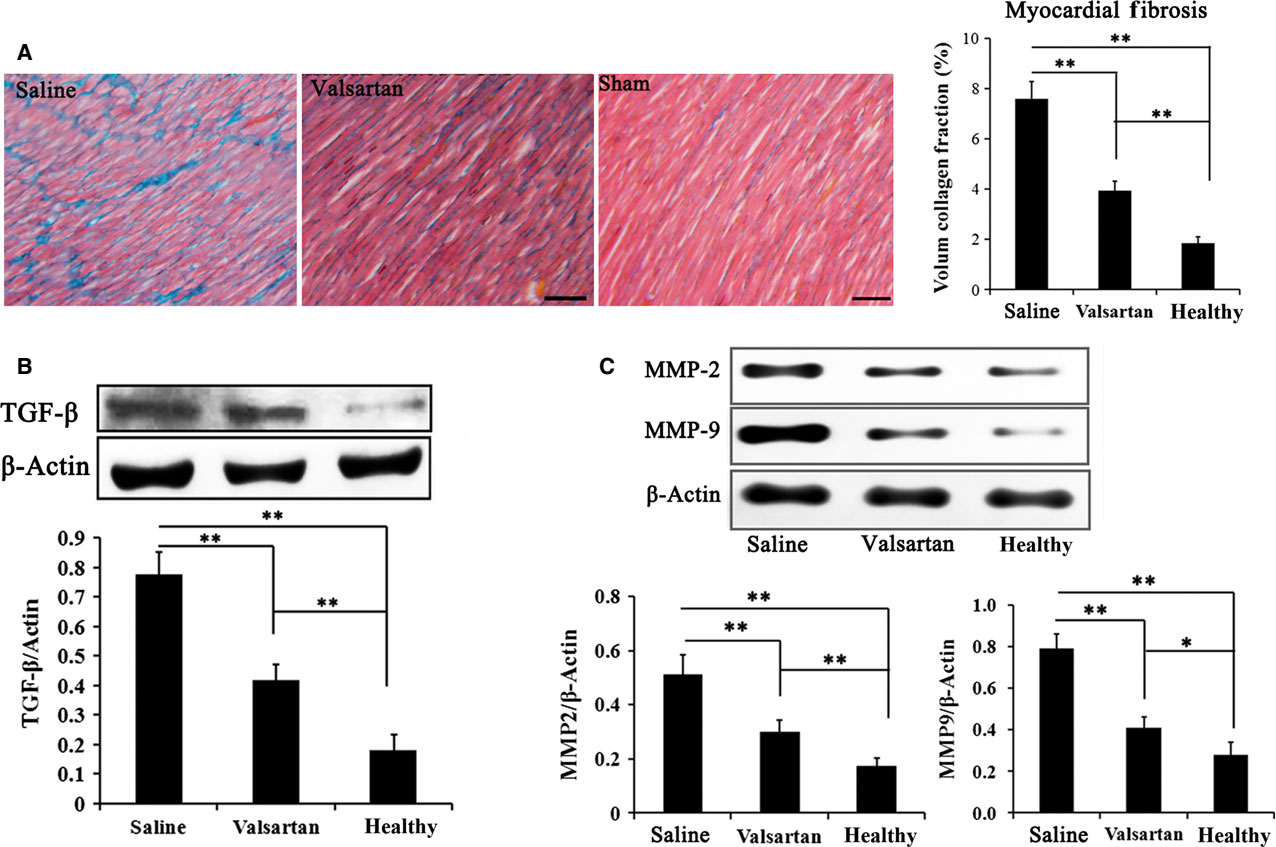
Detection of fibrosis in the border of myocardia infarction and the expression of fibrosis-related proteins (MMP-2 and MMP-9). (A) Four weeks after surgery, Masson’s trichrome staining was performed on heart sections. The border of the infarct zone in each group was observed and it was found that fibrosis in the saline group was significant compared with the normal myocardium, while myocardial fibrosis in the border zone of the infarct was significantly attenuated by valsartan treatment, indicating that application of valsartan inhibits the expansion and progression of myocardial fibrosis after MI (bar = 50 μm). (B) Western blotting analysis showed that Hif-1α and TGF-β level was significantly higher in the saline group compared with normal myocardium, which was lowered because of valsartan treatment. (C) Expression of MMP-2 and MMP-9 was also significantly evaluated in the saline group, and was found to be significantly decreased by valsartan treatment. These results are consistent with myocardial fibrosis in different groups (*n* = 10, *P < 0.05;**P < 0.01).

At 4 weeks, we further analysed myocardial fibrosis in the border of the infarct zone (areas about 1 mm from the infarct zone) to determine whether the effects of valsartan on inhibiting fibrosis expansion resulted from MI. From Masson’s trichrome-stained sections, we observed that the border of infarct zone demonstrated obvious blue staining in the saline-treated group, indicating abundance of collagen in the infarct border zone. However, in the valsartan-treated group, the blue staining area was significantly less than that in the saline group, suggesting that valsartan treatment significantly inhibited the progression or expansion after MI (Fig.4A).

Therefore, we also detected the level of Hif-1α, TGF-β, MMP-2 and MMP-9 with Western blotting in the border of the infarcted zone. As shown in Figure4B, the expressions of Hif-1α, TGF-β, MMP-2 and MMP-9 in the normal myocardium were relatively low, while in the border of the infarct myocardium (saline group), the levels of Hif-1α, TGF-β, MMP-2 and MMP-9 were significantly higher (*P* < 0.01). In the border of the infarct myocardium that received valsartan treatment, though the levels of Hif-1α, TGF-β, MMP-2 and MMP-9 were also significantly increased compared to the normal myocardium, they were much lower than those receiving saline treatment (Fig.4B and C). The results suggest that administration of valsartan inhibited the expression of fibrosis-related proteins TGF-β, MMP-2 and MMP-9, which may reduce the accumulation of collagen and further prevent the enlargement of myocardial fibrosis.

### Cardiac function

**Figure 5:**
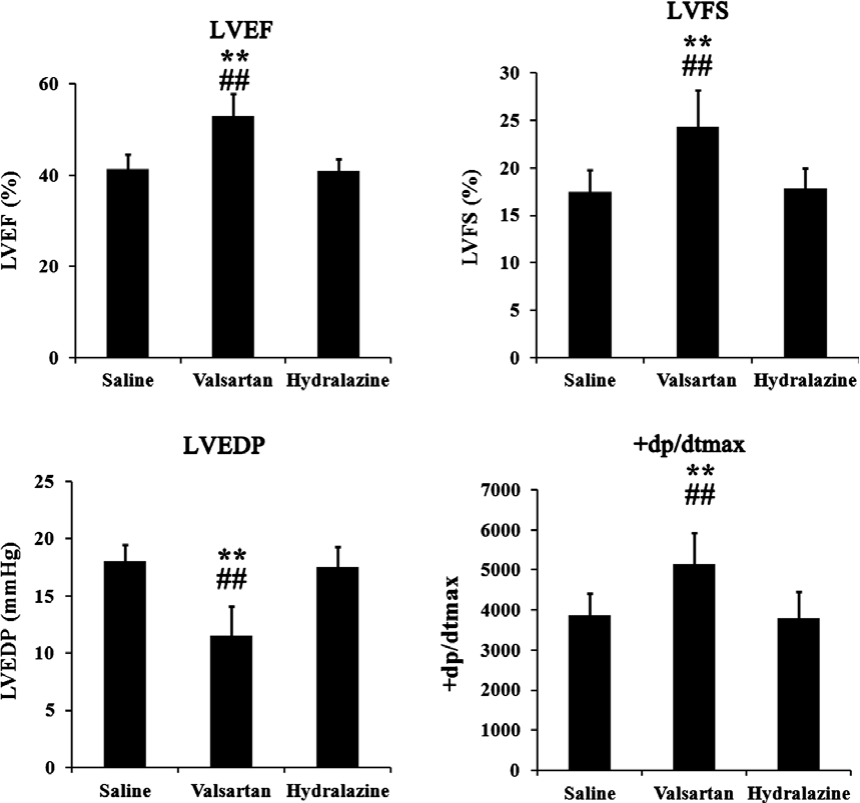
The effects of saline and valsartan on cardiac function were evaluated by echocardiography and haemodynamics. Comparing the groups receiving injection of saline or hydralazine, echocardiography measurement showed that both LVEF and LVFS in rats receiving valsartan injection were significantly increased 4 weeks after surgery. Haemodynamics analysis demonstrated consistent results with that by echocardiography – that LVEDP significantly decreased and +dp/dt_max_ significantly increased in valsartan-treated rats compared to saline- and hydralazine-treated groups, indicating that treatment by valsartan exerted beneficial effects on heart function after MI (*n* = 10, *P < 0.05; **P < 0.01).

Four weeks after surgery, echocardiography was performed to evaluate the effect of valsartan on cardiac function. The results demonstrated that both LVEF and LVFS were significantly improved in valsartan-treated animals compared to saline-treated ones (*P* < 0.01, Fig.5). The functional improvement by valsartan after MI was further confirmed by haemodynamic measurement, which revealed that the injection of valsartan markedly decreased LV end-diastolic pressure (LVEDP) compared to saline (*P* < 0.01). Meanwhile, the maximum LV change in pressure over time (+dp/dt_max_) was significantly enhanced because of the injection of valsartan compared to the injection of saline (*P* < 0.05). Compared with saline treatment, hydralazine treatment did not result in significant improvement in cardiac function.

The above results suggest that the administration of valsartan after MI could exert protective effects on cardiac function after MI, consistent with previous reports, and these protective effects were independent of blood pressure lowering.

### Histological evaluation

**Figure 6:**
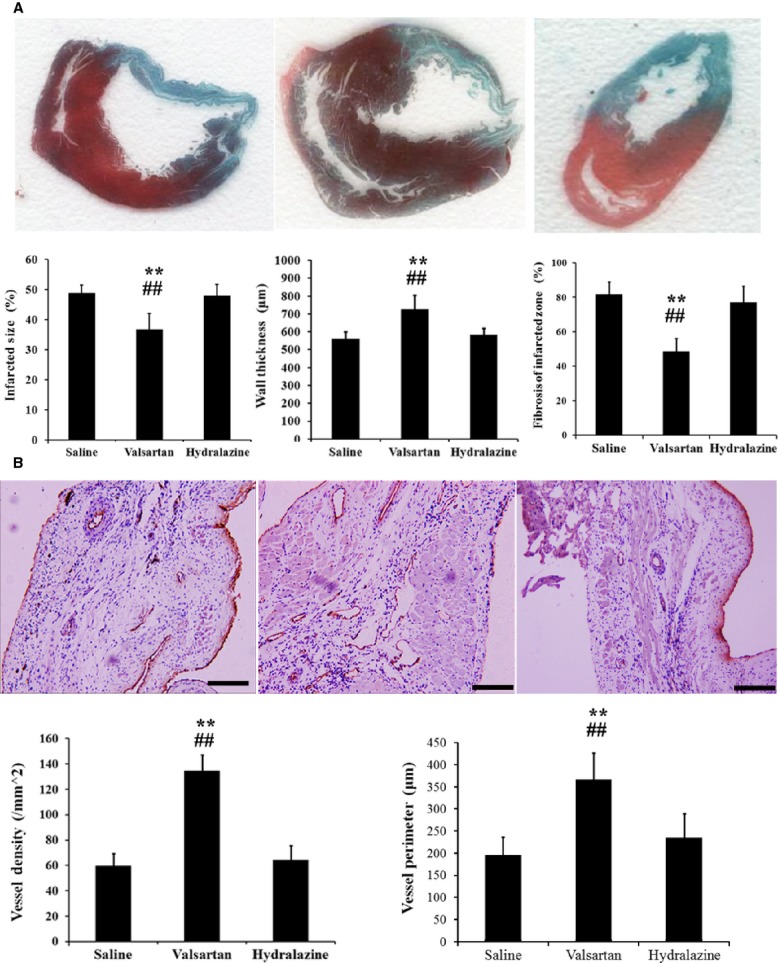
Evaluation of infarct size, wall thickness, myocardial fibrosis and vascular densities. (A) Viable myocardium was stained red and infarct area was stained green by Masson’s trichrome staining. Quantitative analysis showed that both infarct size and myocardial fibrosis in valsartan-treated group were significantly smaller than that in control and hydralazine groups, while wall thickness of infarct zone was significantly larger in the valsartan-treated group compared with that in control and hydralazine groups. (B) Immunostaining against v-WF demonstrated that significantly more tubular structures could be observed in heart sections from valsartan-treated animals than that from saline- and hydralazine-treated groups. Vessel perimeters were also significantly higher in the valsartan-treated group (*n* = 10, **P < 0.01 compared with saline group, ##*P* < 0.01 compared with hydralazine group, bar = 100 μm).

Masson’s trichrome staining of cardiac tissue slices at 4 weeks was performed to evaluate the potential effect of valsartan on infarct size, infarct wall thickness and myocardial fibrosis. As shown in Figure6A, fibroblasts and collagen were largely accumulated in the infarct area after MI. Compared with the saline-treated control group, valsartan treatment significantly prevented the enlargement of infarct size and increased the wall thickness of the infarct zone (*P* < 0.01). In addition, more viable myocardium could be detected in the valsartan-treated group than that in the saline-treated group, indicating that myocardial fibrosis was inhibited by valsartan after MI. Compared with the saline-treated group, hydralazine treatment did not produce significant effects on infarct size, wall thickness and myocardial fibrosis.

### Vascular density and diameter

Following histological evaluation, we performed immunohistochemical analysis to determine neovascularity in the ischaemic myocardium after valsartan administration. The heart sections were immunostained with the anti-vWF antibody to display the vascular structures in infarct areas. The results demonstrated significantly more blood vessels in the valsartan-treated group compared to the saline- and hydralazine-treated groups (Fig.6B). The diameters in the valsartan-treated group were also significantly larger than those in the other groups. The results provide evidence that valsartan promoted neovasculature formation in the ischaemic myocardium, which may be closely related to improved cardiac function.

## Discussion

Fibrosis or scarring formation, which is defined as the accumulation of excess collagen and may finally lead to cardiac malfunction or heart failure, is one of the main pathological progressions after MI. Ang II has been confirmed to play important roles through its type I receptor in the process. It has been demonstrated that ischaemia up-regulated Ang II which further induced TGF-1β/Smad expression, resulting in collagen accumulation. However, whether other mechanisms are involved Ang II-mediated myocardial fibrosis remains unknown. In this study, we demonstrated that Hif-1α pathway also participated in Ang II-mediated myocardial fibrosis after MI. Furthermore, it may have synergetic effects with TGF-1β/Smad in the process, contributing to Ang II-mediated myocardial fibrosis after MI. The findings may provide novel insights into the mechanisms of Ang II-induced cardiac fibrosis and remodelling, as well as into the cardiac protection of valsartan.

The process of fibrosis could be affected by many distinct factors, such as inflammation, fibroblast growth, activation-related microRNAs, etc. TGF-β, MMP-2 and MMP-9 have been extensively involved in the modulation of fibrosis. Furthermore, it has been revealed that TGF-β-related pathways in fibrosis were related to the regulation of differentiation, recruitment, and activation of myofibroblasts that produced and remodelled ECM 23–25. In this study, we confirmed that valsartan significantly regulated the levels of TGF-β, MMP-2 and MMP-9. That valsartan prevents the deposition of collagen and inhibits fibrosis through regulation of TGF-β production has largely been investigated. Typically, valsartan as an antagonist of the angiotensin receptor has been revealed in previous studies to produce protective effects in liver peritoneal fibrosis 8,26, pulmonary injury 27 and LV hypertrophy 28. In the process of fibrosis, Ang II could bind with receptor II to induce the synthesis and reduce the degradation of ECM components. Meanwhile, TGF-β could be induced by Ang II 29–31. In the present study, we observed that valsartan could reduce the expression of TGF-β and its downstream molecular MMP-2 and MMP-9, consistent with previous studies 8,32.

Hif-1α was recently demonstrated to be involved in tissue fibrosis and influenced by Ang II 16,17, such as in vascular and renal fibrosis. However, no previous report has focused on the roles in the process of myocardial fibrosis. In this study, we simultaneously explored the effects of TGF-β and Hif-1α in Ang II-induced myocardial fibrosis and revealed that Hif-1α was another factor in the process. Both TGF-β and Hif-1α were suppressed by valsartan, indicating that they were regulated by Ang II through its AT1 receptor. Although Hif-1α was also somewhat influenced by TGF-β, neither of them could completely restore valsartan-suppressed fibrosis. When both of them were up-regulated, the suppression of valsartan was mostly restored. The results suggest that the TGF-β and Hif-1α pathways may play synergetic roles in myocardial fibrosis, and the cardiac protection by valsartan after MI was through inhibiting both TGF-β and Hif-1α.

In the rat model of MI, we also observed another protective effect of valsartan on cardiac function in addition to inhibiting fibrosis, *i.e*. increased vascularization. It has been known that Hif-1 α is a power transcription factor that increases VEGF expression and vascularization. However, in this study, valsartan reduced the expression of Hif-1 α. This seems contradictory. In fact, it is not such a case. Hif-1α regulated VEGF only in certain conditions, *e.g*. HIF-1α induced VEGF production in pituitary tumour cells 33, and activated HIF-1α promotes VEGF synthesis in mesenchymal stem cells 34. In some other conditions, VEGF expression and angiogenesis were independent of HIF-1α level, *e.g*. under hypoxic conditions,VEGF expression in hepatocellular carcinoma cells was HIF-1-independent, but was primarily controlled by the Akt/PI3K and SP1 pathways 35. Similarly, in chronic moderate hyperoxia in the mouse brain, it was found that HIF-1α accumulation increased, but VEGF expression and microvascular density decreased 36. Therefore, it could be inferred from previous reports that Hif-1α did not always increase VEGF expression and vascularization, as in some hypoxia conditions discussed above. This may explain why inhibited HIF-1α expression in the study did not reduce vascularization. Regarding of myocardial neovascularity, a possible explanation is that the reduction in fibrosis because of valsartan treatment could improve the microenvironment for migration of endothelial cells and thus promote the progression of angiogenesis. It has been demonstrated that the progression of angiogenesis was associated with the haematopoietic microenvironment composed of cell populations, ECM and various cell factors 23. During angiogenesis, the degradation of ECM could promote the migration of endothelial cells 24,25. Therefore, the inhibition of fibrosis by valsartan may assist the migration of endothelial progenitor cells to promote angiogenesis.

In this study, though we demonstrated the synergetic roles of TGF-β1 and HIF-1α in Ang II-mediated myocardial fibrosis after MI, we failed to reveal the underlying mechanisms of Ang II-induced synergistic effects of the TGF-β1 and HIF-1α signalling pathways. This is a limitation of the study. Actually, several questions deserved in-depth investigation, *e.g*. TGF-β1/Smad signalling may have pro-fibrotic and anti-fibrotic effects in dependency on whether Smads are up-regulated or down-regulated. Furthermore, whether and how angiogenesis and VEGF expression were regulated independent of HIF-1α changes need to be researched? Addressing these issues may provide novel insights into the mechanisms of Ang II-mediated collagen accumulation and changes in myocardial vasculature, and these will be the emphasis of our future work.

In summary, we confirmed that both TGF-β and Hif-1α played important roles in Ang II-mediated myocardial fibrosis after MI. They may play synergetic roles in the process. Administration of valsartan attenuated myocardial fibrosis through inhibiting both TGF-β and Hif-1α pathways. Our study may provide novel insights into the mechanisms of Ang II-induced cardiac fibrosis and remodelling, as well as into the cardiac protection of valsartan.

## Disclosure

None declared.
